# B7-H3 (CD276) regulates myeloid-derived suppressor cell differentiation in pulmonary fibrosis

**DOI:** 10.3389/fimmu.2026.1903767

**Published:** 2026-08-10

**Authors:** Francina Gonzalez De Los Santos, Alyssa Rosek, Akira Ando, Sem H. Phan, Navin R. Mahadevan, Tianju Liu

**Affiliations:** 1 Department of Pathology, University of Michigan Medical School, Ann Arbor, MI, United States; 2 Graduate Program in Immunology, University of Michigan Medical School, Ann Arbor, MI, United States; 3 Department of Respiratory Medicine, Nagoya University Hospital, Nagoya, Japan

**Keywords:** B7-H3, fibroblasts, fibrosis, MDSC (myeloid-derived suppressor cells), myeloid cells

## Abstract

**Introduction:**

Myeloid-derived suppressor cells (MDSCs) are elevated in cancer, chronic inflammation and fibrosis. The immune checkpoint molecule B7-H3 (CD276) is upregulated in fibrotic conditions and has been implicated in the expansion and recruitment of MDSCs to the fibrotic lung; however, the underlying mechanism remains unclear.

**Methods:**

To investigate the role of B7-H3 in MDSC induction, myeloid differentiation and lung fibrosis, we employed *in vivo* B7-H3 blockade, cell type-specific deletion mouse models, exogenous B7-H3 administration, and *in vitro* bone marrow differentiation assays.

**Results:**

Our results showed that *in vivo* B7-H3 blockade, and cell type-specific deletion of B7-H3 in collagen-expressing or myeloid cells attenuated lung fibrosis. Reduction of B7-H3 was associated with decreased MDSC expansion and increased numbers of MHCII^+^ F4/80^+^ cells. In contrast, exogenous B7-H3 administration *in vivo* reduced MHCII^+^ F4/80^+^ cells in the lung. *In vitro*, treatment of bone marrow cells with B7-H3 promoted MDSC generation and inhibited myeloid differentiation. Additionally, we found that soluble B7-H3, potentially cleaved from fibroblasts by matrix metalloproteinases (MMPs), contributed to the induction of MDSCs in co-cultured bone marrow cells and the suppression of myeloid cell differentiation. Finally, CD84, a novel immunosuppressive marker for M-MDSCs, expressed on MDSCs was required for B7-H3-mediated enhancement of their immunosuppressive capacity toward T cells.

**Discussion:**

Together, these findings reveal that bleomycin-induced B7-H3 signals to the bone marrow, skewing myeloid differentiation toward an immature MDSC phenotype by inhibiting normal myeloid maturation during fibrosis.

## 1 Introduction

Myeloid-derived suppressor cells (MDSCs) are increased significantly in cancer and chronic inflammatory conditions (1–7) but are largely absent in healthy tissues. Characteristically, MDSCs are known primarily for their immunosuppressive activity, both directly on innate and adaptive immune responses, and indirectly by their capacity to recruit T-regulatory cells (7, 8). MDSCs represent a heterogeneous population of cells, broadly classified as either granulocytic MDSCs (G-MDSCs) or monocytic MDSCs (M-MDSCs) based on their morphology, cell surface markers, and presumed precursor cell of origin. In addition to the phenotypical identification, their ability to suppress immune cells is a defining characteristic of MDSCs (2). MDSCs originate from common myeloid progenitors (CMPs) which give rise to granulocyte-monocyte progenitors (GMPs). GMPs can differentiate into myeloblasts (MBs) that develop into G-MDSCs and neutrophils, or into monocyte-dendritic cell progenitors (MDPs) that give rise to M-MDSCs and monocytes (4, 5, 9). A large body of evidence highlights the critical role of MDSCs in the tumor microenvironment, where MDSC-mediated immunosuppression contributes to tumor initiation, progression and metastasis (10–16).

Beyond cancer, numerous studies have reported elevated levels of circulating MDSCs in chronic pathological conditions, including persistent infection, inflammation, and tissue injury or remodeling (1, 3, 17–21). In patients with idiopathic pulmonary fibrosis (IPF), increased circulating MDSC frequencies correlate with progressive lung function decline (19, 20). Evidence from animal models further supports a role for MDSC activation in lung fibrosis; notably, studies in mice demonstrate that telomerase reverse transcriptase and B7-H3-dependent pathways promote MDSC expansion and recruitment from bone marrow (BM) during the initiation and progression of lung fibrosis (20).

The immune checkpoint molecule B7-H3 (CD276) is a member of the B7 family of lymphocyte ligands, originally characterized for its role in regulating lymphocyte function. Early studies have reported conflicting findings, describing both stimulatory and inhibitory effects on immune responses (22–27). Subsequent research has demonstrated that B7-H3 contributes to immunosuppression within the tumor microenvironment (27–30). More recently, B7-H3 has been implicated in the expansion and recruitment of immature myeloid cells, including MDSCs, to tumor sites and fibrotic lesion areas (7, 18, 20, 31–33–35). B7-H3 expression is upregulated in several pathological conditions, such as cancer and lung fibrosis (18, 22, 27, 30, 36, 37) and, in cancer, elevated expression correlates with poor prognosis (38). In patients with idiopathic pulmonary fibrosis (IPF), increased levels of plasma soluble B7-H3 (sB7-H3) are associated with declining lung function, enhanced immunosuppressive activity, and acute disease exacerbation (18, 20, 36, 37). Analysis of publicly available single-cell RNA sequencing data (IPF Cell Atlas, GEO accession: GSE128033) reveals significant upregulation of B7-H3 expression in fibroblasts, myofibroblasts, and macrophages from IPF lungs (20). Our previous findings show that B7-H3 can activate mouse BM-derived M-MDSCs and promote lung fibroblast expression of collagen I and α-smooth muscle actin (α-SMA) (20). These results suggest that BM-derived MDSCs may exert a paracrine effect on myofibroblast differentiation during pulmonary fibrosis through B7-H3 mediated signaling (20). Notably, antibody-mediated neutralization of B7-H3 in bleomycin (BLM)-induced lung fibrosis reduces recruitment of MDSCs to the lung and suppresses early inflammation and fibrosis (20).

Despite these insights, the mechanisms governing MDSC differentiation during fibrosis remain poorly understood. Some evidence suggests that skewing myeloid differentiation toward the MDSC lineage may result from blockade of normal myeloid differentiation pathways (39). Chronic or prolonged cytokine stimulation may also contribute to the pathological activation of MDSCs (18, 20, 40, 41). However, the precise mechanisms underlying MDSC emergence in fibrotic disease remain unclear. We hypothesize that B7-H3 may mediate BM MDSC differentiation, recruitment, and/or activation in response to lung injury. In the present study, we investigated the potential relationship between B7-H3 and myeloid cell differentiation, including both mature myeloid cells and immature MDSCs, and explored how fibroblast or myeloid-derived B7-H3 regulates MDSC differentiation and contributes to pulmonary fibrosis.

## 2 Materials and methods

### 2.1 Human subjects

Peripheral blood samples were obtained from 63 patients with IPF and from normal controls recruited at the University of Michigan Medical School hospital. Plasma samples from normal controls were collected from 10 healthy volunteers. All IPF patients had a confirmed diagnosis and met the diagnostic criteria established by the American Thoracic Society and the European Respiratory Society (42). Detailed information on patient demographics and blood sample processing has been described previously (20).

### 2.2 Mice and animal model of pulmonary fibrosis

Female C57BL/6J mice (6–8 weeks old stock no. 000664), ColCre (C57BL/6J-Tg [Col1α2-Cre-ER (T)], stock no. 029567), and male LysMCre mice (B6.129P2-Lyz2tm1(cre)Ifo/J, stock no. 004781) were purchased from Jackson Laboratory (Bar Harbor, ME, USA). LysMCre mice were backcrossed to wild type (WT) C57BL/6J mice for at least six generations. A floxed B7-H3 (Cd276) mouse line on a C57BL/6J background was a generous gift from Dr. Brad St. Croix (National Cancer Institute, Frederick, MD, USA). PD1fl/fl, LysMCre^+^/^-^ (LysMCre-PD1) knockout KO mouse line was a generous gift from Dr. Vassiliki A. Boussiotis (Beth Israel Deaconess Medical Center and Dana-Farber/Harvard Cancer Center, Boston, MA, USA). To generate inducible collagen-expressing or myeloid cell specific B7-H3 (KO) mice, floxed B7-H3 mice were crossed with ColCreERT2^+^/^-^ or LysMCre^+^/^-^ mouse, respectively. The genotype of B7-H3fl/fl, ColCreERT2^+^/^-^ or LysMCre^+^/^-^ mice were confirmed by PCR, and the resulting B7-H3fl/fl, ColCreERT2^+^/^-^ conditional KO (ColCre-B7-H3 cKO), or B7-H3fl/fl, LysMCre^+/-^ myeloid-specific (LysM-B7-H3) KO mice were used for the bleomycin model of pulmonary fibrosis studies. Six to ten week old female ColCre-B7-H3 cKO or control ColCreERT2^+^/^-^ mice; LysMCre-B7-H3 KO or LysMCre control mice (referred to as WT for simplicity) were used to induce pulmonary fibrosis with bleomycin (BLM) (Mead Johnson, Princeton, NJ, USA) by oropharyngeal instillation on day 0 at a dose of 2.25 units/kg body weight as described previously (43). The numbers of mice included were 7–10 mice/BLM group and 5 mice/control group. For the inducible ColCre-B7-H3 cKO or ColCreERT2^+^/^-^ mice, 1 mg/mouse/daily of tamoxifen (Sigma, St. Louise, MO, USA) was injected into the animals for 10 consecutive days prior to BLM treatment, and daily tamoxifen injection was continued for 5 doses/week after BLM injection until the day of euthanasia. Animals were randomly assigned to the indicated treatment groups (n = 5~10 mice each group). Mice were anesthetized intraperitoneally with ketamine (87 mg/kg) and xylazine (10 mg/kg). For euthanasia, an overdose of ketamine (120 mg/kg) and xylazine (10 mg/kg) was administered intraperitoneally, followed by exsanguination or vital organ removal as a secondary method. The mice were euthanized on day 21 after BLM treatment and the lungs were harvested rapidly for tissue processing, lung single cell isolation, hydroxyproline assay, or histopathological examination. Where indicated, the blocking antibodies against mouse B7-H3 (clone MJ18, Cat# BE0124, Bio X cell, Lebanon, NH, USA) or the isotype rat IgG1 (0.3 mg/mouse) were injected into the mice via tail veins starting on day 7 after BLM treatment. Recombinant mouse B7-H3 (rmB7-H3) (R & D Biosystems, Minneapolis, MN, USA) was injected into mice via tail vein (0.15 mg/kg) on day 0, 2, and 4, and then the mice were euthanized on day 4 or day 21. Brequinar (BRQ, R & D Biosystems) was injected starting on day 1 (10 mg/kg/daily i.p.) for 20 days. Two to three independent biological experiments were performed for each *in vivo* study.

### 2.3 Cell isolations

Mouse lung fibroblasts (MLFs) were isolated using collagenase III and DNase I (Worthington Biochemical Crop., Lakewood, NJ, USA), and maintained in DMEM supplemented with 10% plasma-derived fetal bovine serum (PDS; Animal Technologies, Tyler, TX, USA), 10 ng/ml of EGF, and 5 ng/ml of PDGF (R&D systems, Inc. Minneapolis, MN, USA) as before (44). The whole lung single-cell suspension was prepared using collagenase III and DNase I and then resuspended in the flow buffer (PBS containing 1% BSA) for flow cytometry (44). Whole mouse BM cells flushed from femurs and tibias were maintained in RPMI-1640 (Thermo Fisher Scientific, Waltham, MA, USA) containing 10% fetal bovine serum (Sigma) and analyzed by flow cytometry. For the indicated experiments, the cells were stimulated by 10 ng/ml of Granulocyte Macrophage Colony-Stimulating Factor (GM-CSF, R & D Systems) for 2 days, and then treated with 2 µg/ml rmB7-H3 (R & D Systems) in the presence of BRQ (2 µM) for another 36 hours, or pre-treated with rmB7-H3 for 24 hours, and followed by 72 hours of GM-CSF treatment. Mouse BM CD11b^+^ Gr1^+^ MDSCs were isolated using EasySep™ Mouse Myeloid-Derived Suppressor Cell Isolation kit (STEMCELL Technologies, Cambridge, MA, USA) according to the manufacturer’s instructions.

### 2.4 Flow cytometry analysis

All antibodies were purchased from Biolegend (San Diego, CA, USA) unless otherwise specified. Mouse whole lung single-cell suspension or BM cells were stained with CD45-PECy7 (clone 30-F11, Cat# 103114), CD11b-FITC (clone M1/70, Cat# 101206), Ly6C-APC (clone HK1.4, Cat# 128016), Ly6G-BV421 (clone 1A8, Cat# 127628), F4/80-ACPCy7 (clone BM8, Cat# 123118), MHCII-BV510 (clone M5/114.152, Cat# 107636), CD11c-AF488 (clone N418, Cat# 117311), CD84-PE (clone mCD84.7, Cat# 122806), and PD1-APCCy7 (clone 29F.1A12, Cat# 135223). M-MDSCs and G-MDSCs were defined as CD11b^+^ Ly6C^hi^ Ly6G^-^ and CD11b^+^ Ly6C^-/lo^ Ly6G^+^, respectively. Macrophage-like cells were defined as MHCII^+^ F4/80^+^ or CD11b^+^ F4/80^+^ and Myeloid-like cells were defined as CD11c^+^. Cells were also stained with antibody cocktail containing lineage-FITC (Cat# 133302), CD150-BV510 (clone TC15-12F12.2, Cat# 115929), CD34-PECy7 (clone HM34, Cat# 128618), cKit-BV785 (clone 2B8, 105841) or cKit-PE (clone 104D2, Thermo Fisher Cat#12-1178-42), Sca1-PerCP (clone D7, Cat# 108121), CD16/32-BV421 (clone 93, Cat#101331), PD1-APCCy7 and CD84-PE. Granulocyte-monocyte progenitors (GMPs) were defined by lineage^-^ CD150^-^ CD34^+^ cKit^+^ Sca1^-^ CD16/32^+^. Term “GMP-like cells” was used in the indicated experiments where CD16/32 was not included for progenitor analysis. CD8-APC (clone 53-6.7, Cat# 100712) and CD4-BV421 (clone GK1.5, Cat# 100437) were used for CD8^+^ or CD4^+^-T cell population as indicated. Human whole white blood cells were stained with B7-H3-PE (clone DCN70, Cat# 331606), CD4-APC (clone A161A1, Cat# 357408), CD25-APCCy7 (clone BC96, Cat# 302614) and Foxp3-AF488 (clone MF-14, Cat# 126405). For human MDSC analysis, peripheral blood mononuclear cells (PBMCs) were isolated from patients with IPF using BD VacutainerTM (CPT) mononuclear cell preparation tube (Thermo Fisher Scientific, Cat# 02-685-125). Isolated PBMCs were plated in 6-well plates at a density of 3 × 10^6^ cells per 2ml per well overnight and then treated with 2µg/ml recombinant human B7-H3 (rhB7-H3, R & D Systems) for 3 days. MDSC analysis by flow cytometry was performed as previously described (20). The following antibodies were purchased from Biolegend: HLA-DR –BV421 (clone L243, Cat# 30763), CD33-APCCy7 (clone P67.6, Cat# 366614), CD11b-PE (clone M1/70, Cat# 101207), CD14-FITC (clone 63D3, Cat# 367116), CD15-PECy7 (clone HI98, Cat#301906). Human M-MDSCs were defined as HLA-DR- CD33^+^ CD11b^+^ CD14hi CD15- and human G-MDSC were defined as HLA-DR^-^ CD33^+^ CD11b^+^ CD14^-/lo^ CD15^+^. MLFs were stained with a-SMA-FITC (clone 3F9, Cat# bsm-33187M-FITC, Bioss Antibodies (300 Tradecenter Dr # 4610, Woburn, MA 01801)). All flow cytometry data were acquired with a NovoCyte flow cytometer and analyzed by NovoExpress software (ACEA Biosciences, Inc., San Diego, CA, USA) or FlowJo10.10 as indicated in the respective figure legends.

### 2.5 Transwell co-culture

Co-culture of bone marrow (BM) cells with primary MLFs (passages 2-5) isolated from WT or ColCre-B7-H3 cKO mice was performed using 6-well transwell systems with 0.4 µm pore membranes (BD Bioscience, San Jose, CA, USA) as previously described (20). Where indicated, WT MLF were pre-treated with Matrix Metalloproteinases Inhibitor (MMPI), GM6001 (10 µM; Selleckchem, Houston, TX, USA) for 24 hours, followed by TGFβ treatment (10 ng/ml) for 48 hours prior to co-culture. BM cells were harvested 48 hours after co-culture for flow cytometry analysis.

### 2.6 Cell migration assay

Cell migration assays were performed using FluoroBlok™ 96-Multiwell Insert Plates with 3.0 µm membrane (#351161, Thermo Fisher Scientific) as previously described (18). CD11b^+^Gr1^+^ MDSCs were pre-stained with 5 μM Calcein AM (564061, BD Bioscience) and incubated with anti-Tlt2 antibodies (10 µg/ml, clone# 656906, Cat# MA5-24295, Thermo Fisher Scientific) or isotype IgG control for 1 hour prior to plating. The cells were cultured with the conditioned media (CM) collected from control or Transforming Growth Factor Beta (TGFβ) treated MLFs (10 ng/ml for 48 hours). After 18 hours of incubation, fluorescence was measured using a SpectraMax Gemini EM Microplate Reader (Molecular Devices, San Jose, CA, USA).

### 2.7 T cell suppression assay

BM-derived MDSCs were isolated using EasySep™ Mouse Myeloid-Derived Suppressor Cell Isolation kit (STEMCELL Technologies, Cambridge, MA, USA) as previously described (20). Where indicated, MDSCs were pre-treated with rmB7-H3 (2 µg/ml) in the presence of GM-CSF (10 ng/ml) for 3 days, followed by incubation with CD84 blocking antibodies (10 µg/ml; Cat# TAB-359LC (E), Creative Biolabs, Shirley, NY) or IgG control for 2 hours before co-culture. Freshly isolated splenocytes were activated with CD3/28 Dynabeads (Thermo Fisher Scientific) and IL-2 (R & D Systems). The activated splenocytes were stained with CFSE, and then co-cultured with pre-treated MDSCs at the indicated MDSC: T cell ratios. Other experimental controls include un-activated T cell alone without or with CFSE staining; CD3/28+IL2 activated T cell alone without or with CFSE staining. The cells were harvested 3 days later and stained with Cell viability dye-eFluor 780 (Thermo Fisher Scientific, Cat# 65-0865-14), CD4-BV421 (clone GK1.5, Cat# 100437, BioLegend) and CD8-APC antibodies. The intensity of CFSE within CD4^+^ or CD8^+^ populations was analyzed by flow cytometry.

### 2.8 Hydroxyproline assay and histopathological analysis

Whole lung collagen content was assessed by measuring hydroxyproline levels in lung homogenates, previously described (45, 46). Results were expressed as percentage change in hydroxyproline content of the right lungs. For fibrosis evaluation, lungs were inflated with 10% buffered formaldehyde via intratracheal perfusion and fixed for 24 hours. Lung tissues were then paraffin-embedded, sectioned, and stained with Masson’s trichrome.

### 2.9 Real time PCR and Western blotting analysis

For quantitative mRNA analysis, total RNA was isolated from lung tissue, fibroblasts, or sorted MDSC. The Taqman primer/probes of the following genes were purchased from Thermo Fisher, Waltham, MA, USA): mouse α-SMA (Acta2), type I procollagen (Col1a1), TGFβ (Tgfb), B7-H3, B7-H1 (Cd274), IL6 (Il6), Stat3 (Stat3), C/EBPβ (Cebpb), PD1 (Pdcd1), Arg1 (Arg1), Nos2 (Nos2). For each qPCR assay, 50 ng of total RNA was used with RNA-to-Ct 1 step kit (Thermo Fisher Scientific). 18S rRNA served as an internal control. Results were expressed as 2^–ΔΔCT^ as previously described (47). The antibodies used for Western blotting included anti-mouse α-SMA (Cat# 2547, Sigma), anti-collagen I (bs-10423R, Bioss Antibodies, Woburn, MA, USA), and anti-B7-H3 (Cat# BE0124, Bio X cell).

### 2.10 B7-H3 ELISA assay

The levels of soluble B7-H3 (sB7-H3) in human or mouse plasma samples were measured using ELISA kits (LS Bio Sciences, Inc.), in accordance with the manufacturer’s protocol.

### 2.11 Statistics

All data are expressed as mean ± S.D. unless otherwise indicated. Differences between means of treatment and control groups were assessed for statistical significance using one-way ANOVA followed by Tukey’s multiple comparisons test. Student’s t-test was used for the comparison between two groups. Associations between variables were evaluated by linear regression and Pearson correlation. These statistical analyses were performed using GraphPad Prism (version 9.5.1, GraphPad Software, San Diego, CA, USA). A p value less than 0.05 was considered significant. The following symbols were used to denote levels of significance in all figures: * p < 0.05, ** p < 0.01, *** p < 0.005, **** p < 0.0001 in all figures. p values greater than 0.05 were not labeled.

### 2.12 Study approval

All human studies were reviewed and approved by the Institutional Review Board at the University of Michigan, and informed consent was obtained from each patient or volunteer. All animal studies were reviewed and approved by the Institutional Animal Care and Use Committee at the University of Michigan.

## 3 Results

### 3.1 B7-H3 deficiency in collagen-expressing cells reduces bleomycin-induced lung fibrosis, M-MDSCs and increases MHCII^+^ F4/80^+^ macrophage-like cells

**Figure 1:**
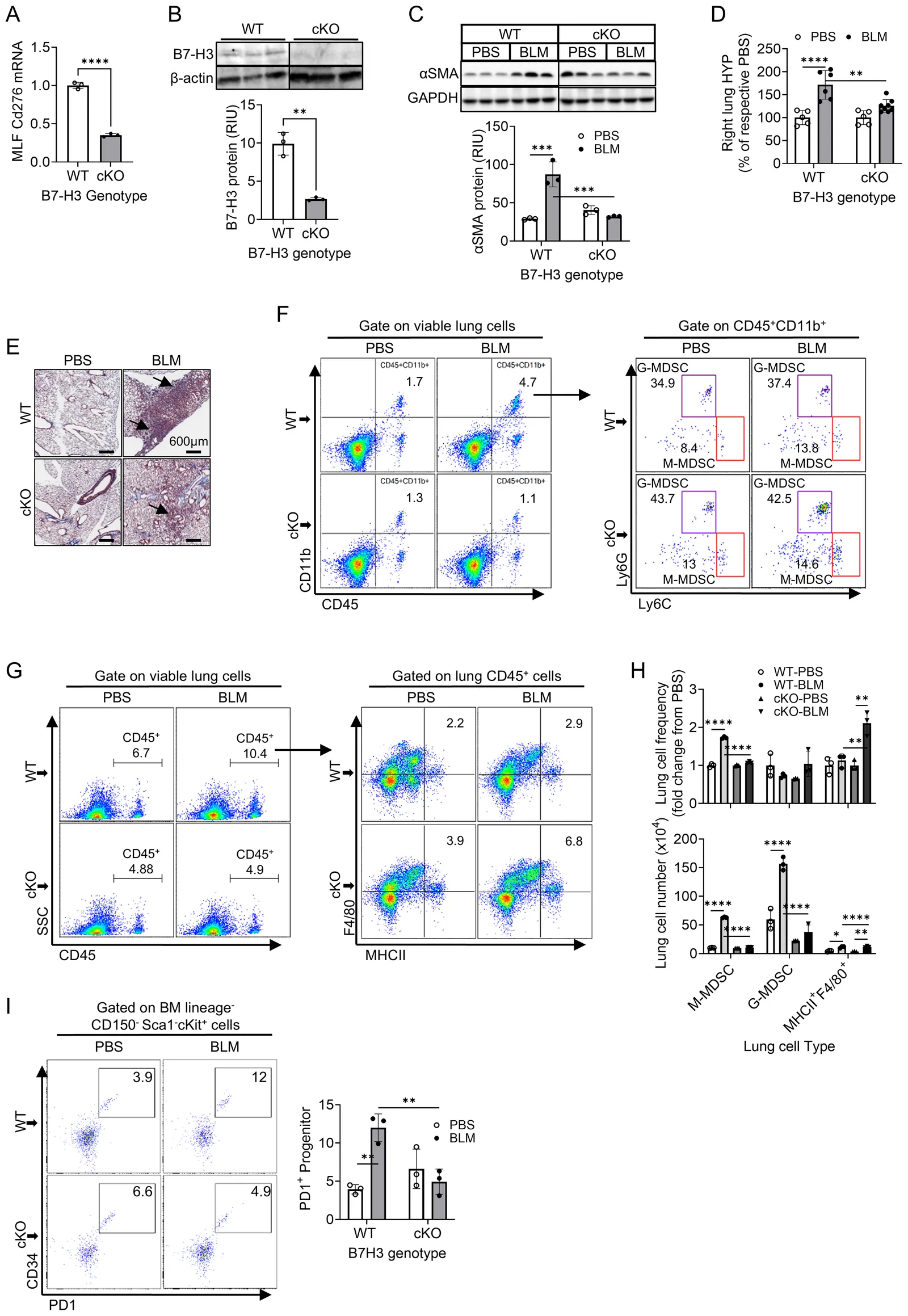
B7-H3 deficiency in collagen-expressing cells attenuates BLM-induced lung fibrosis and M-MDSCs. One mg/mouse/daily of tamoxifen was injected into the ColCre-B7-H3 cKO and ColCre (WT) for 10 consecutive days prior to BLM treatment, and daily tamoxifen injection was continued for 5 doses/week after BLM injection until the day of euthanasia at day21. **(A)** MLF from WT and cKO were isolated at day 10 after tamoxifen treatment and were analyzed for B7-H3 mRNA by qPCR. **(B)** Lung tissue lysates were collected after tamoxifen treatment and were analyzed for B7-H3 protein by Western blotting (top, ~ 20 kDa of B7-H3 bands are shown on the representative blots) and quantification is shown below. The predicted glycosylated forms of B7-H3 at ~90–100 kDa were analyzed as shown in **Supplementary Figure 1A**. **(C)** Lung tissue lysates were isolated from controls and BLM treated lungs at day 21 and were analyzed for α-SMA protein by Western blotting. The representative blots **(top)** and quantitative analysis (bottom) are shown. **(D)** Right lung homogenates were analyzed for hydroxyproline content. **(E)** Lung tissue histopathology was examined by Masson’s trichrome staining. Scale bar: 600 µm. **(F)** Lung single-cell suspensions were analyzed for MDSCs by flow cytometry. The gating strategy of lung CD45^+^ CD11b^+^ cells in single cell suspension is shown in **Supplementary Figure 1C**. The CD45^+^ CD11b^+^ population was subsequently analyzed for bone marrow (BM)-derived M-MDSCs and G-MDSCs, defined as CD45^+^ CD11b^+^ Ly6C^hi^ Ly6G^-^ and CD45^+^ CD11b^+^ Ly6C^-/lo^ Ly6G^+^, respectively. **(G)** The lung CD45^+^ population was further analyzed for MHCII^+^ F4/80^+^macrophage-like cells. Representative plots are shown. **(H)** Quantitative analyses of panels **(F)** and **(G)**. The absolute cell numbers of lung M-MDSCs, G-MDSCs, and MHCII^+^ F4/80^+^ populations were calculated based on total lung cell counts and corresponding cell frequencies. **(I)** BM cells isolated from B7-H3 cKO or WT mice were analyzed for PD1^+^ GMPs by flow cytometry. The BM cells were first analyzed for lineage markers and CD150. The lineage^-^ CD150^-^ population was then separated by Sca1 and cKit. Sca1^-^cKit^+^ population that is shown in **Supplementary Figure 1D** was further gated for CD34^+^ and PD1^+^ cells. The lineage^-^ CD150^-^ Sca1^-^ cKit^+^ CD34^+^ population was further confirmed as CD16/32^+^ GMPs (**Supplementary Figure 1E**). N = 3–8 mice per group. In **(I)**, each data point represents pooled BM cells from 2 mice. At least two independent mice experiments were performed. Student’s test was performed in 1A, 1B; one-way Anova analysis in 1C, 1D, 1F, and 1I. *p 0.05, **p 0.01, ***p 0.005, ****p 0.0001.

Single-cell RNA sequencing data (** IPF Cell Atlas **, GEO accession GSE128033) (20) indicate that lung fibroblasts and myofibroblasts are the major sources of B7-H3 induced in IPF. To investigate the role of B7-H3 specifically in fibroblasts, we used tamoxifen-induced ColCre-B7-H3 conditional knockout (cKO) mice, in which B7-H3 is selectively deleted in Col-expressing fibroblasts. We first verified that fibroblasts isolated from ColCre-B7-H3 cKO lungs exhibited reduced B7-H3 mRNA expression compared with fibroblasts from WT mice (**Figure 1A**) and that total lung B7-H3 levels were markedly lower in ColCre-B7-H3 cKO mice relative to WT controls (**Figure 1B**; **Supplementary Figure 1A**). Following BLM treatment, survival rates were comparable between WT (7 out of 10, 70%) and ColCre-B7-H3 cKO mice (8 out of 11, 73%), with no statistically significant difference (**Supplementary Figure 1B**). Loss of B7-H3 in ColCre-B7-H3 cKO mice reduced lung α-SMA protein expression on day 21 after BLM challenge compared with approximately 3-fold induction observed in WT mice (**Figure 1C**), suggesting a decrease in fibroblast differentiation. Consistently, a lung hydroxyproline assay indicated that BLM-induced collagen accumulation in WT mice was significantly attenuated in ColCre-B7-H3 cKO mice, decreasing from 1.7 to 1.2-fold increase relative to PBS-treated controls (**Figure 1D**). Histopathology analysis supported these findings: Masson’s trichrome staining showed fewer and more scattered lesions in ColCre-B7-H3 cKO lungs compared with the extensive fibrotic lesions observed in WT mice (**Figure 1E**), indicating fibroblast B7-H3 deficiency ameliorates lung fibrosis. We next examined the abundance of MDSC and macrophage-like cells during the initiation of lung fibrosis in control and cKO mice by flow cytometry. BLM treatment led to a 1.64-fold increase in BM-derived M-MDSC (13.8 vs. 8.4%) in WT mice, whereas ColCre-B7-H3 cKO mice displayed elevated basal M-MDSCs that remained unchanged following BLM installation (**Supplementary Figure 1C**; **Figures 1F and 1H**). In contrast to M-MDSCs, a significant increase in BM recruited macrophage-like cells (CD45+ MHCII+ F4/80+) was observed in cKO mice after BLM treatment, as opposed to the decline to near control levels seen in WT mice at day 21 (**Figure 1G**, 1H).We further investigated MDSC progenitor cells in the BM. Interestingly, PD1-expressing progenitors, potentially GMPs, were induced by BLM about 3-fold in the BM of WT mice (12 vs 3.9%), which was essentially abolished in B7H3 cKO mice (**Supplementary Figure 1D** and **Figure 1I**). To confirm and distinguish GMP from common myeloid progenitor progenitors (CMP), the additional GMP marker CD16/32 was also analyzed, and the results confirmed that BLM-induced GMPs in WT BM were reduced in B7-H3 cKO mice (s**Figure 1E**). Collectively, these results indicate that fibroblast-specific B7-H3 deficiency mitigates BLM-induced lung fibrosis, reduces BM-derived M-MDSC expansion, and promotes macrophage-like cell differentiation. These effects were closely associated with the reduced frequency of PD1+ GMP BM progenitors.

### 3.2 Fibroblast-derived sB7-H3 might induce BM-MDSC differentiation

**Figure 2:**
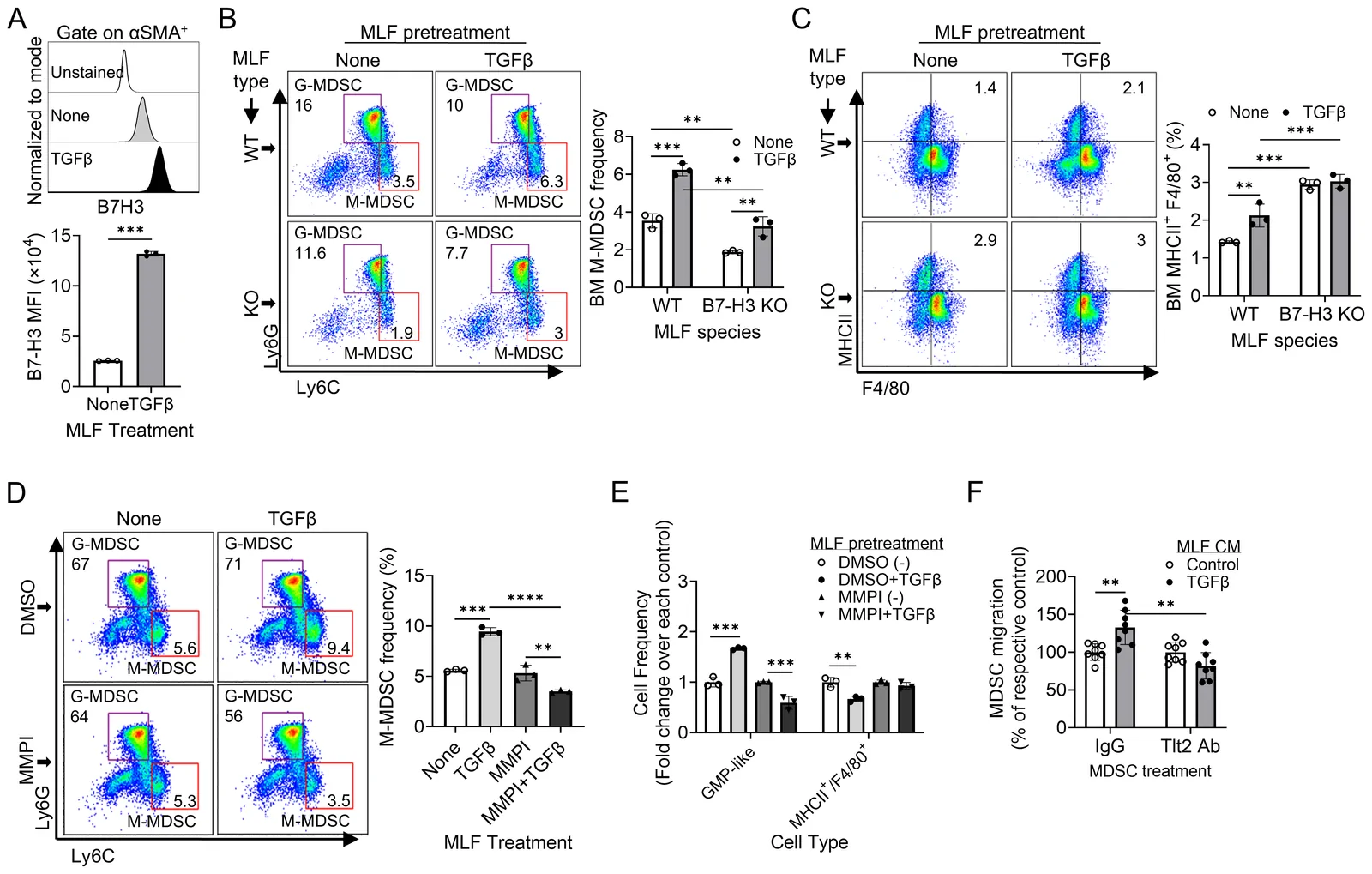
Lung fibroblast-derived sB7-H3 induces BM-MDSC differentiation and inhibits MHCII^+^ F4/80^+^macropahge-like cell differentiation. **(A)** Primary MLF cultures, with or without TGFβ stimulation, were analyzed for B7-H3 expression in α-SMA expressing fibroblast by flow cytometry and analyzed by FlowJo. Representative flow plots **(top)** and quantitative analysis (bottom) are shown. MLFs isolated from WT or ColCre-B7-H3 cKO mice were passaged 3–5 times (α-SMA-positive cells accounted for 92.6% of total MLFs; see **Supplementary Figure 2**) and pretreated with TGFβ, and then co-cultured with naïve BM cells in a transwell system. After 48 hours of co-culture, BM cells were analyzed by flow cytometry for MDSCs **(B)** and MHCII^+^ F4/80^+^ cells **(C)** using same gating strategies as in **Figure 1F, G**. The representative plots and quantification are shown. **(D)** Wild type MLFs were pretreated with an MMPI 24 hours prior to 24 hour TGFβ stimulation and then co-cultured with BM cells. After 24 hours of stimulation, BM cells were analyzed for MDSCs by flow cytometry. Representative flow plots and quantitative data for M-MDSCs are shown. **(E)** GMP-like progenitors (lineage^-^ CD150^-^ Sca1^-^ cKit^+^ CD34^+^) and MHCII^+^ F4/80^+^ cells were analyzed by flow cytometry and quantitative data are shown. **(F)** The Calcein AM prelabeled CD11b^+^ Gr1^+^ MDSCs were incubated with anti-Tlt2 antibodies before being placed in the upper inserts of a 3 µm transwell system. Conditioned media (CM) from control or TGFβ treated MLF were added to the lower chambers. The fluorescence intensity in the lower chamber was measured after 18 hours of incubation. Results are expressed as percentage of the activity of TGFβ-CM relative to control CM. N = 3-8. Student’s test was performed in 2A; one-way Anova analysis in 2B-2F. *p 0.05, **p 0.01, ***p 0.005, ****p 0.0001.

Based on our findings that fibroblast-derived B7-H3 contributes to lung fibrosis and increased M-MDSC accumulation, we next assessed the effect of fibroblast-derived B7-H3 on BM MDSC induction. Cell surface membrane B7-H3 expression on α-SMA-positive mouse lung fibroblasts (MLFs) was confirmed by flow cytometry and found to be significantly elevated following Transforming Growth Factor Beta (TGFβ) stimulation of myofibroblast differentiation (**Figure 2A**; **Supplementary Figure 2**). MLFs were then isolated from wild-type (WT) mice or from mice lacking B7-H3 in fibroblasts (ColCre-B7-H3 cKO). After pre-treatment with TGFβ, these fibroblasts were co-cultured with freshly isolated BM cells. BM cells co-cultured with TGFβ-treated WT fibroblasts exhibited a higher frequency of M-MDSCs compared with untreated fibroblasts (6.3% vs. 3.5%). Similar to the observation in BLM-treated ColCre-B7-H3 cKO lung (**Figure 1F**), G-MDSC frequency was not significantly affected. Notably, M-MDSC induction was significantly reduced when BM cells were co-cultured with ColCre-B7-H3 cKO fibroblasts with or without TGFβ (3.5% vs. 1.9% and 6.3 vs 3, respectively) (**Figure 2B**). Additionally, similar to previous *in vivo* experiments (**Figure 1G**), MHCII+ F4/80+ macrophage-like cells were increased in BM cells co-cultured with ColCre-B7-H3 cKO fibroblasts, both with and without TGFβ stimulation, compared with WT fibroblasts (**Figure 2C**). These results suggest that fibroblast-derived B7-H3 possibly promotes BM-derived MDSC generation while inhibiting differentiation of macrophage-like cells. To examine whether this effect could be mediated by sB7-H3 cleaved from the fibroblasts, we pre-treated fibroblasts with a broad-spectrum matrix metalloproteinase inhibitor (MMPI) known to suppress sB7-H3 release (48) prior to co-culture with BM cells in a transwell system. The TGFβ-stimulated fibroblasts increased M-MDSCs frequency from 5.6% to 9.4%; and this effect was completely abolished by MMPI pre-treatment, reducing M-MDSCs to 3.5% (**Figure 2D**). Similar to the observation in fibrotic lung between WT and ColCre-B7-H3 cKO mice (**Figure 1F, H**), G-MDSCs were not significantly affected by TGFβ or MMPI (**Figure 2D**). The increase in MDSC progenitors likely GMPs by TGFβ-stimulated fibroblasts was similarly abrogated by MMPI treatment whereas levels of MHCII+ F4/80+ cells, which were decreased following TGFβ treatment, were unaffected by MMPI (**Figure 2E**). Previous studies have shown that sB7-H3 interacts with the myeloid cell-like transcript 2 receptor (Tlt2) to mediate the chemotactic activity of MDSCs (18, 20). To test whether fibroblast-derived sB7-H3 could act as a chemoattractant via TLT2 signaling, we conducted a chemotaxis assay using BM-derived MDSCs. Conditioned medium from TGFβ-activated fibroblasts significantly enhanced chemotactic activity toward BM-MDSCs. This chemotactic effect was markedly reduced when MDSCs were pretreated with anti-TLT2 antibodies (**Figure 2F**). Collectively, these findings indicate that activated fibroblasts serve as a source of sB7-H3 that may contribute to the activation, expansion, and migration of BM-derived M-MDSC, while simultaneously inhibiting the differentiation of MHCII+ F4/80+ macrophage-like cells.

### 3.3 B7-H3 regulates the induction of MDSC and CD11c^+^ myeloid-like cell maturation in BM

**Figure 3:**
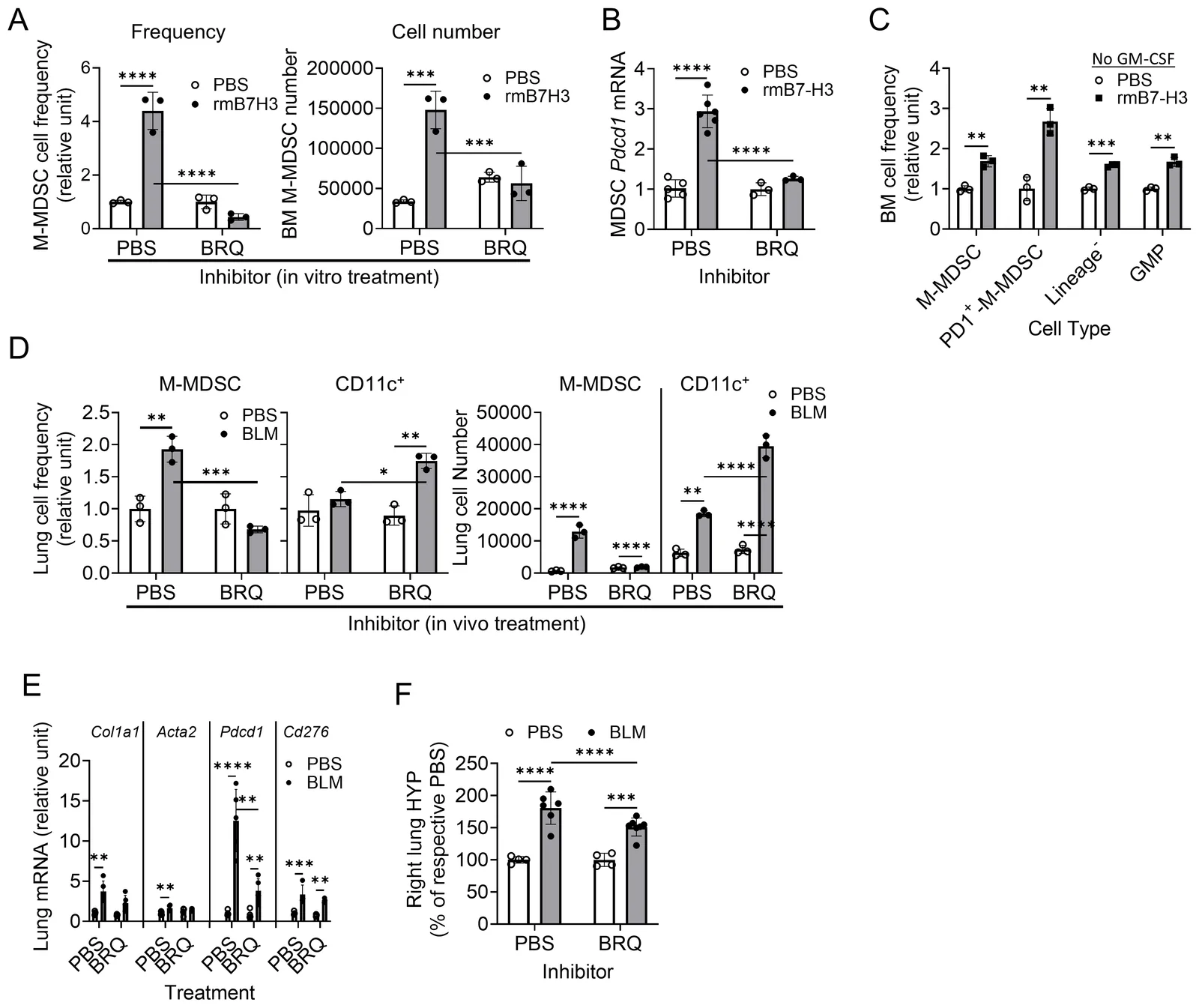
B7-H3 induces BM-MDSC differentiation by inhibiting CD11c^+^myeloid-like cell differentiation during lung fibrosis. **(A)** Freshly isolated naïve BM cells were first expanded by GM-CSF (10 ng/ml) and then treated with PBS or BRQ (2 µM) for 24 hours, followed by rmB7-H3 treatment (2 µg/ml) for an additional 36 hours. Quantitative data for M-MDSCs analyzed by flow cytometry are shown as fold change (left panel) and absolute number per of 1 ×10^6^ cells (right panel). **(B)** BM-derived MDSCs were isolated after BRQ and rmB7-H3 treatments and were analyzed for PD1 (Pdcd1) mRNA expression by qPCR. **(C)** The indicated cell populations, including CD16/32^+^ GMPs, were analyzed after rmB7-H3 treatment in the absence of GM-CSF, flow cytometry quantification is shown. The y-axis represents changes relative to their respective control. **(D)** BLM was administered into 6–8 weeks old C57BL/6J WT female mice on day 0, followed by daily intraperitoneal injection of BRQ until day 20 and lungs were harvested on day 21. Single-cell suspension from lungs of PBS or BLM-treated mice with or without BRQ treatment were analyzed by flow cytometry for M-MDSDC (left) and CD11c^+^ cells (right). Their absolute numbers are shown (right panel). **(E)** Lung tissues RNA was analyzed for the indicated mRNAs by qPCR. **(F)** Lung tissue lysates were assessed for hydroxyproline content. N = 3-8. One-way Anova analysis was performed in all. *p 0.05, **p 0.01, ***p 0.005, ****p 0.0001. The animal experiments were performed twice.

MDSCs arise from GMPs that normally give rise to mature myeloid cells. B7-H3 might expand M-MDSCs during fibrosis via its effect on GMPs (**Figure 1I**). To explore the potential impact of B7-H3 on myeloid cell maturation, we investigated the effects of B7-H3 on Brequinar (BRQ)-induced MDSC differentiation and myeloid cell maturation. BRQ, a potent and selective inhibitor of dihydroorotate dehydrogenase by inhibition of pyrimidine synthesis and cell growth, has been shown to inhibit MDSC differentiation while enhancing myeloid maturation in breast cancer (39, 49). Isolated BM cells were treated with recombinant mouse B7-H3 (rmB7-H3) with or without concomitant BRQ treatment following GM-CSF expansion. Flow cytometry analysis showed that rmB7-H3 increased M-MDSC frequency by 4-fold in BM cells compared with untreated cells in the absence of BRQ, confirming that rmB7-H3 can cause MDSC polarization. However, the induction was completely abolished in the presence of BRQ. The changes in M-MDSC absolute number were consistent with the frequency changes (**Figure 3A**). To explore the immunosuppressive capacities of MDSC, we measured PD1 mRNA (Pdcd1) in the sorted BM MDSCs (CD11b+ Gr1+). PD1 mRNA was highly induced by rmB7-H3 (3-fold) and was markedly reduced in the presence of BRQ (**Figure 3B**). In the unexpanded/undifferentiated population without GM-CSF treatment, rmB7-H3 treatment induced the lineage-negative population, MDSC progenitor GMPs, and PD1-expressing M-MDSCs (**Figure 3C**). These indicate a potential role for B7-H3 in regulation of myeloid cell development at the early progenitor stage. We then sought *in vivo* validation of these findings in the BLM-induced lung fibrosis model. In BLM-treated groups, 6 out of 7 mice in PBS group, and 6 out of 8 mice in BRQ group survived 21 days after BLM treatment, which was not statistically different between two groups (s**Figure 3A**). Daily BRQ injections caused reduction in both MDSC frequency and the cell number with a concomitant increase in myeloid-like cells as indicated by increased CD11c+ cells after BLM treatment (**Figure 3D**), along with a slight insignificant decrease in total cell number of BRQ-treated lung compared with PBS control group at day 21 after BLM induction (s**Figure 3B**). While BLM treatment in the control group caused significant increases in mRNA levels of collagen I (Col1a1) and α-SMA (Acta2) as expected, BRQ-treated mice showed only insignificant or no increase in these mRNA expressions following BLM treatment (**Figure 3E**). Consistent with its *in vitro* effect on PD1 expression, the lung PD1 mRNA upregulation in BLM treated mice was markedly reduced in BRQ-treated mice, without affecting BLM-induced B7-H3 expression significantly (**Figure 3E**). BLM-induced lung fibrosis, assessed by lung hydroxyproline, was significantly reduced in BRQ-treated mice compared with the control group (**Figure 3F**). These findings suggest that elevated level of B7-H3 in lung fibrosis (18, 20) may be capable of inducing MDSCs, especially PD1-expressing M-MDSCs, while inhibiting myeloid-like cell differentiation/maturation in the fibrotic injured lung (**Figure 1H** and **Figure 3D**).

### 3.4 Myeloid cell-specific B7-H3 deficiency inhibits induction of MDSC and promotes MHCII^+^ F4/80^+^ macrophage-like cell differentiation

**Figure 4:**
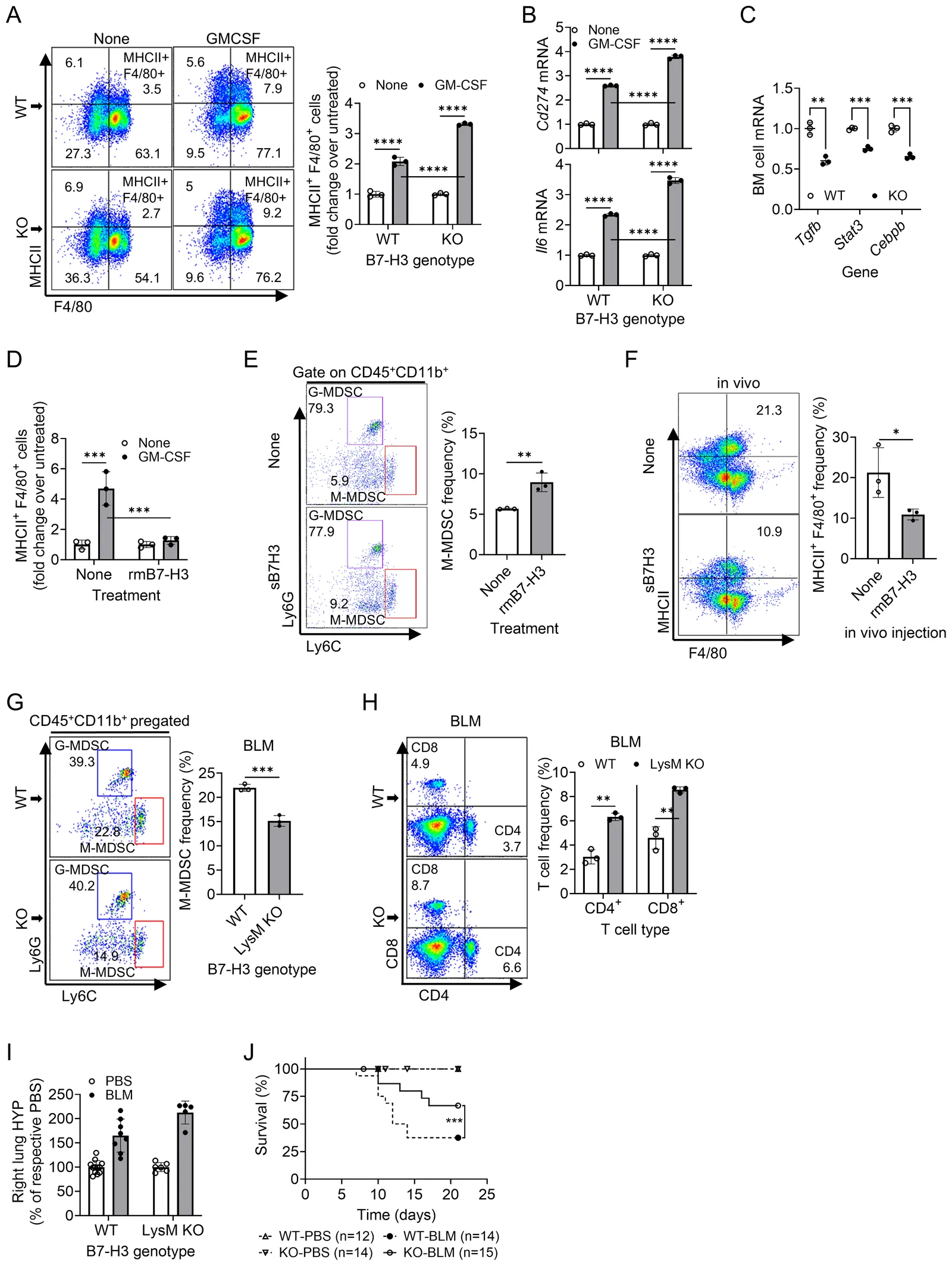
Myeloid B7-H3 deficiency attenuates MDSC induction and stimulates MHCII^+^ F4/80^+^ macrophage-like cell differentiation. BM cells were isolated from LysMCre (WT) or LysMCre-B7-H3 KO mice and treated with PBS or 10 ng/ml GM-CSF for 2 days. **(A)** BM cell flow cytometry analysis and representative flow plots (left) and quantitative data showing fold changes (right) are presented. **(B)** GM-CSF-induced gene expression of indicated genes in WT and KO BM cells was analyzed by qPCR. **(C)** Basal expression levels of indicated genes in untreated WT and KO BM cells were analyzed by qPCR. **(D)** Naïve mouse BM cells were pretreated with rmB7-H3 for 24 hours, followed by 10ng/ml GM-CSF treatment for an additional 72 hours. The same gating strategy as in **(A)** was used to identify MHCII^+^ F480^+^ macrophage-like cells. **(E)** Wild type mouse BM-derived (CD45^+^ CD11b^+^) MDSCs were treated with 2μg/ml rmB7-H3 for 24 hours and analyzed by flow cytometry (left) and quantitative data are shown. The gating strategy and parent populations of CD45^+^ CD11b^+^ cells are described in **Figure 1F**. **(F)** C57BL/6J WT mice were treated *in vivo* with three intravenous doses of 0.15 mg/kg rmB7-H3 on day 0, 2, and 4. Lung MHCII^+^ F4/80^+^ macrophage-like cells were analyzed by flow cytometry on day 21 after the final injection. The gating strategy is same as in 1G. **(G)** BLM was administrated to 6–10 weeks old female LysMCre control (WT) or LysM-B7-H3 KO mice at day 0. Lung single cell suspension was isolated at day 21 and were analyzed by flow cytometry for MDSCs. The gating strategy and parent populations of CD45^+^ CD11b^+^ cells are described in **Figure 1F**. **(H)** Lung CD4^+^ or CD8^+^-T cells from viable cell populations were analyzed by flow cytometry. **(I)** Lung fibrosis was evaluated by measuring hydroxyproline levels in lung tissue lysates. Data were combined from two independent experiments and expressed as percentage relative to PBS controls. **(J)** Survival was assessed on day 21 following BLM or PBS treatment. N = 3–4 mice per group in all *in vivo* experiments, representative flow cytometry plots are shown in **(A, E, F, G)** and **(H)** All experiments were performed at least twice. Student’s test was performed in **(C, E, F)**; one-way Anova analysis in **(A, B, D, H, I)**. *p 0.05, **p 0.01, ***p 0.005, ****p 0.0001.

To further explore whether B7-H3 can affect MDSC differentiation in a cell autonomous manner during fibrosis, we generated mice lacking B7-H3 in myeloid cells using LysMCre-B7-H3 KO mice. To confirm depletion of CD276 in LysMCre-B7-H3 KO myeloid cells, we isolated CD45+, CD45-, MDSCs and F4/80+ cells and measured CD276 mRNA levels. LysMCre-B7-H3 KO CD45+, MDSCs and F4/80+ cells expressed less CD276 mRNA than controls (**sFigure 4A**). We then investigated whether loss of B7-H3 affects myeloid cell differentiation and MDSC induction *in vitro* using BM cells isolated from LysMCre-B7-H3 KO mice. Myeloid cell-specific B7-H3 depletion significantly enhanced GM-CSF-induced differentiation into MHCII+ F4/80+ macrophage-like cells compared with B7-H3 expressing WT BM cells (3.4-fold vs. 2.3-fold induction, **Figure 4A**), which was associated with increased Il6 and Cd274 mRNA levels (**Figure 4B**). Conversely, markers characteristic of MDSCs, including Tgfβ, Stat3, and Cebpβ, were significantly reduced in LysMCre-B7-H3 KO cells compared with WT BM cells (**Figure 4C**), indicating that loss of B7-H3 in myeloid cells promotes GM-CSF-driven myeloid cell differentiation. In contrast, pre-treatment of BM cells with rmB7-H3 inhibited GM-CSF-induced differentiation of MHCII+ F4/80+ macrophage-like cells. The GM-CSF-induced increase in macrophage-like cells (approximately 4.3-fold) was reduced to about 1.2-fold when BM cells were pre-treated with rmB7-H3 (**Figure 4D**). We used CD11b+ and F4/80+ markers in addition to MHCII+ F4/80+ to further capture macrophage-like cell heterogeneity and define MDSC populations. Similar to the changes in MHCII+ F4/80+ cells, GM-CSF induced an approximately 1.9-fold increase in CD11b+ F4/80+ cells, which was diminished by B7-H3 pre-treatment (**sFigure 4B**). In addition, BM cells treated with rmB7-H3 showed a significant increase in CD11b+ M-MDSCs consistent with our previous observations (**Figure 4E**). We next investigated the role of B7-H3 in myeloid differentiation and lung fibrosis *in vivo*. WT mice treated with rmB7-H3 *in vivo* showed a significant decrease (~50%) in lung MHCII+ F4/80+ macrophage-like cells at day 5 (s**Figure 4C**), and this reduction lasted up to day 21 (**Figure 4F**). Absence of B7-H3 in myeloid cells in LysMCre-B7-H3 KO mice significantly reduced M-MDSCs by 34% (from 22.8% to 14.9%, **Figure 4G**) but failed to affect G-MDSCs after BLM treatment compared with WT cells. This reduction in M-MDSCs was inversely associated with increased lung CD4+ and CD8+ T-cells (1.9-fold and 1.7-fold, respectively) in BLM-treated KO compared with WT mice (**Figure 4H**), consistent with the predominant immunosuppressive role M-MDSCs on T cells (20). The effect of myeloid-B7-H3 deficiency on lung fibrosis was assessed by hydroxyproline measurement, which showed no significant difference in lung collagen content between the WT and LysMCre-B7-H3 KO mice at day 21 after BLM treatment (**Figure 4I**), indicating that loss of B7-H3 in myeloid cells alone is insufficient to alter the progression to late stage BLM-induced lung fibrosis. However, survival was significantly improved in LysMCre-B7-H3 deficient mice compared with WT controls after BLM injury (**Figure 4J**). Together, these data suggest that B7-H3 promotes MDSC differentiation by blocking macrophage-like cell differentiation, likely at an early stage in fibrosis, without directly altering late-stage lung fibrosis.

### 3.5 Blockade of CD84 dampens B7-H3-mediated activation of MDSC immunosuppressive capacity

**Figure 5:**
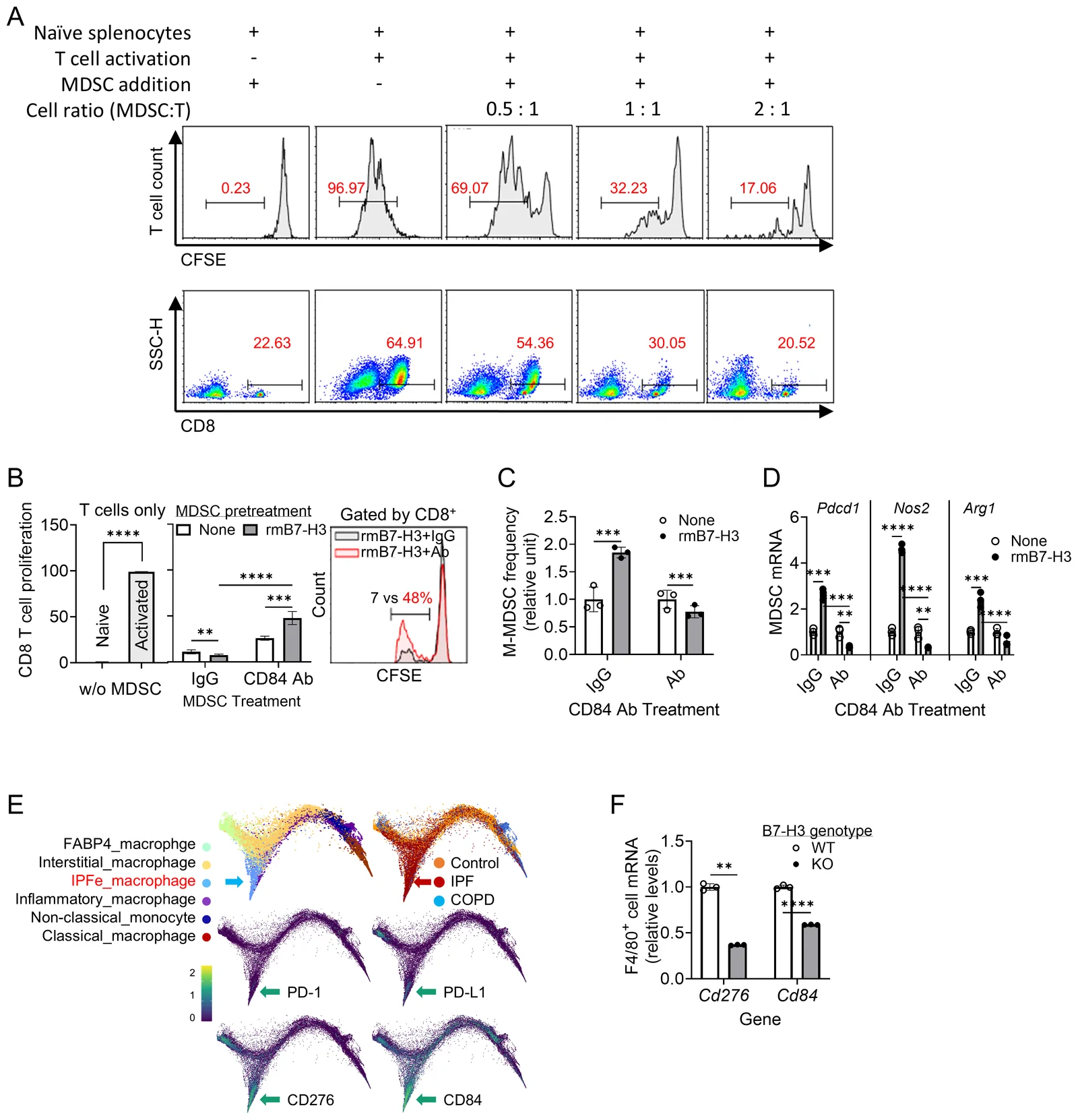
Immunosuppression of T-cell activation is mediated by B7-H3 and CD84. **(A)** T cell suppression assays were performed by co-culturing CFSE pre-stained, activated splenocytes with BM-derived MDSCs at varying MDSC: T cell ratios as indicated above the flow cytometry plots. Cells were harvested 3 days later. CFSE intensity within CD8^+^ T cell populations was analyzed by flow cytometry. The top panel shows CFSE intensity in splenocyte before and after activation, and after addition of MDSCs at different ratio, and the bottom panel shows the frequency of CD8^+^ T cell within the CFSE^+^ population. **(B)** Mouse CD11b^+^ Gr1^+^ MDSCs were treated with rmB7-H3 in the presence of GM-CSF and then incubated with CD84 blocking antibodies or IgG control for 2 hours before the addition of CFSE prelabeled, activated splenocytes. The splenocytes were harvested 3 days later and analyzed for CD8 and CFSE by flow cytometry, and Y-axis indicates percentage of proliferating CD8^+^-T cells in CFSE-strained splenocytes without co-culture (left) or co-cultured with MDSCs (right). A representative overlaid plot of rmB7-H3-treated groups with CD84 antibodies or IgG is shown in the right panel. **(C)** GM-CSF expanded BM cells were preincubated with CD84 blocking antibodies or IgG control prior to rmB7-H3 treatment and then analyzed for M-MDSC by flow cytometry, and quantitative data are shown. **(D)** CD11b^+^ Gr1^+^ MDSCs were isolated after the same treatment as in **(C)** and analyzed for the indicated genes by qPCR. **(E)** Single-cell RNA sequencing data for the indicated genes in lung macrophage populations were extracted from the IPF Cell Atlas (GEO accession GSE136831). IPF e-macrophages are indicated as IPF expanded macrophage. **(F)** F4/80^+^ cells were isolated from LysMCre-B7-H3 KO lung cell suspension using MACS system with F4/80-microbeads, and Cd276 and Cd84 mRNA levels were measured by qPCR. N = 3. One way Anova analysis was performed in all. **p 0.01, ***p 0.005, ****p 0.0001.

CD84 (also known as SLAMF5), is a membrane glycoprotein and member of the signaling lymphocyte activation molecule (SLAM) family. Recently, it has been identified as immunosuppressive marker for MDSCs, particularly for M-MDSCs. CD84-expressing M-MDSCs are specifically induced by B7-H3 in the BM (20, 50). To determine whether CD84 mediated the ability of B7-H3 to enhance MDSC suppression of T cell proliferation, we performed antibody blockade experiments. First, the immunosuppression effect of MDSC on CD8+ T cell proliferation was shown to be dependent on MDSC number, with progressively stronger suppression observed as the MDSC:T cell ratio increased (0.5:1, 1:1, and 2:1) resulting in 16.3%, 53.3%, and 68.4% decreases in CD8+ T cell proliferation, respectively (**Figure 5A**). Next, sorted MDSCs were incubated with CD84 blocking antibodies before co-culture. Spleen CD8+ T cells were robustly activated by CD3/28 plus IL-2 treatment, as shown by the increase in proliferating CD8+ T cells from 0.82 to 99% (**Figure 5B**, left panel). This proliferation was markedly reduced to 11% when CD8+ T cells were co-cultured with MDSCs (at ratio of 1:1) and further decreased to 7.4% in the presence of rmB7-H3. Pre-incubation of MDSCs with CD84 blocking antibodies significantly reversed these inhibitory effects, with a greater restoration of T cell proliferation co-cultured with rmB7-H3-treated MDSCs (7.4 vs 48%) than in controls (11 vs 25%) (**Figure 5B**, middle and right panels). The viability of MDSCs was checked by flow cytometry using cell viability dye after CD84 antibody or IgG treatment, and similar percentages of viable MDSCs between CD84 and IgG treatment were observed (96.3% vs. 95.9%, in **Supplementary Figure 5**). Consistently, rmB7-H3-induced M-MDSC frequencies were markedly reduced in BM cells following CD84 blockade (**Figure 5C**). The B7-H3-induced upregulation of immunosuppressive M-MDSC signature genes, PD1 (Pdcd1), Nos2 and Arg1, was also abolished in sorted MDSCs (CD11b+ Gr1+) after CD84 blockade (**Figure 5D**). Analysis of human single-cell RNA sequencing data from IPF lung macrophages (IPF Cell Atlas, GEO accession GSE136831) revealed strong co-localization of CD84 with B7-H3, PD1 and PD-L1 transcripts (**Figure 5E**). Similarly, mouse CD84 mRNA expression was significantly reduced in F4/80+ cells isolated from LysMCre-B7-H3 knockout mice compared to controls (**Figure 5F**), suggesting that B7-H3 expression is associated with maintaining CD84 levels in myeloid populations. Taken together, these results indicate that the ability of B7-H3 to enhance MDSC-mediated suppression of T cells depends, at least in part, on CD84 expression on MDSCs, positioning CD84 as a functional contributor to B7-H3-driven MDSC immunosuppressive function (20, 50).

### 3.6 Myeloid cell PD1 contributes to B7-H3 induction of BM-MDSC differentiation

**Figure 6:**
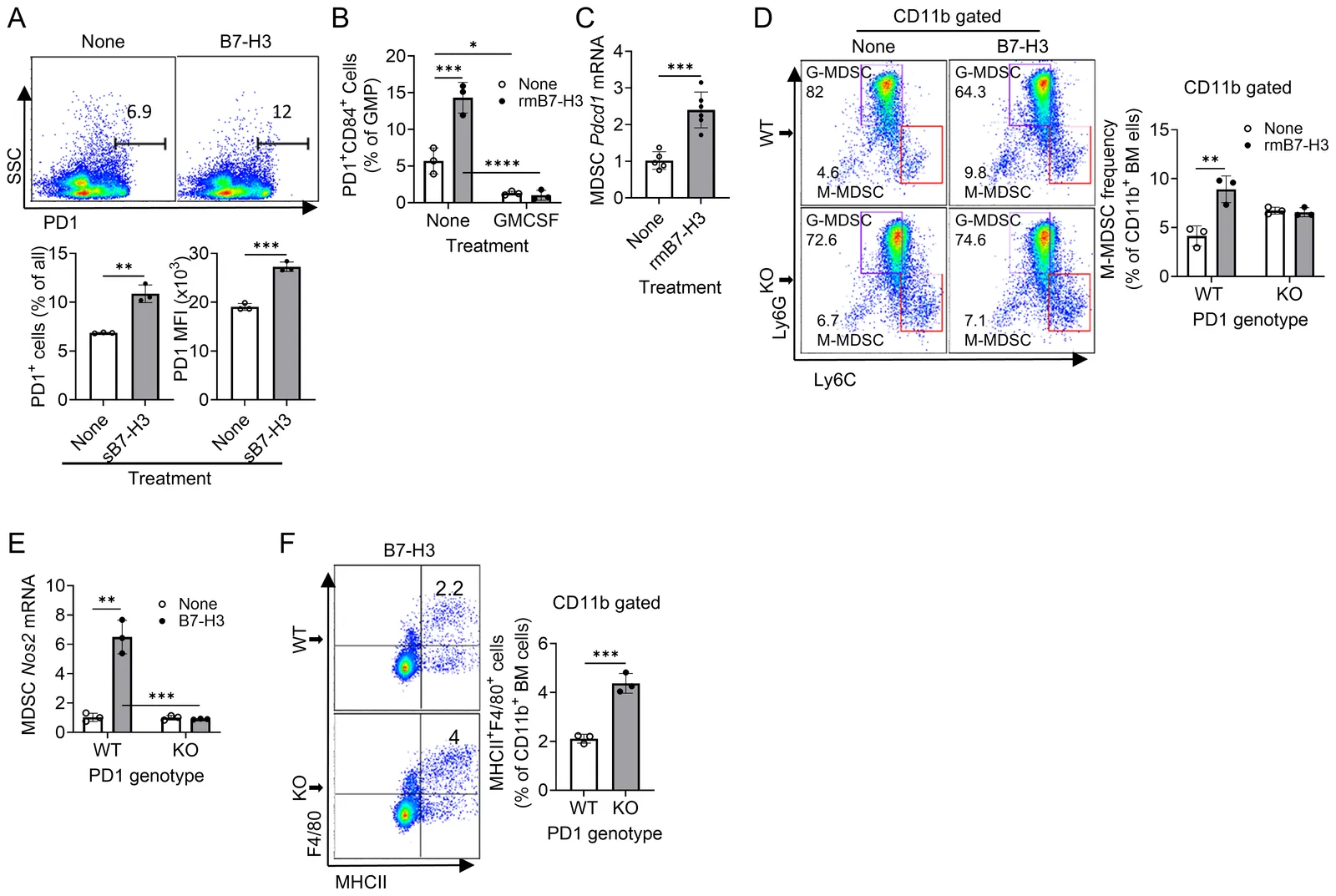
LysM-PD1 ablation in myeloid cells inhibits B7-H3- induced M-MDSC differentiation. **(A)** Freshly isolated BM cells were treated with 2 µg/ml of rmB7-H3 for 24 hours and analyzed for PD1 expression by flow cytometry (top). The quantitative analysis of PD1 frequency and mean fluorescence intensity (MFI) are shown (bottom). **(B)** BM cells were treated with 2 µg/ml of rmB7-H3 in the presence of 10 ng/ml GM-CSF for 36 hours, and then PD1^+^CD84^+^ cells within the GMP population were analyzed by flow cytometry. Quantitative data are shown. **(C)** Sorted mouse CD11b^+^Gr1^+^ MDSCs after the same treatment as in **(A)** were analyzed for PD1 (Pdcd1) mRNA by qPCR. **(D)** BM cells were isolated from LysM-PD1 KO or LysMCre (WT) mice and treated with 2 µg/ml rmB7-H3 for 48 hours. BM MDSCs were analyzed by flow cytometry. **(E)** CD11b^+^ Gr1^+^ MDSCs were sorted from BM cells after treatment with rmB7-H3 in **(D)** and analyzed for Nos2 mRNA by qPCR. **(F)** The BM cells from the rmB7-H3-treated groups were also analyzed for myeloid-like cells in CD11b^+^ BM cell population. N = 3-6. The experiments were repeated three times. A representative flow cytometry plot is shown in **(D)**, **(F)** and **(G)**. Student’s test was performed in **(A, C, F)**; one-way Anova analysis in **(B, D, E)**. *p 0.05, **p 0.01, ***p 0.005, ****p 0.0001.

Recent studies indicate that PD1 expression in MDSC progenitor GMPs is increased in tumor-bearing mice, peaking at an early stage of tumor inoculation, and that myeloid cell-specific PD1 ablation prevents the accumulation of GMPs and skews myeloid lineage fate (51). Given the fact that B7-H3 was able to increase GMP and PD1+ M-MDSCs in undifferentiated BM cells (**Figure 3C**), we investigated whether B7-H3 modulates PD1 expression in BM cells. We isolated BM cells, treated them with rmB7-H3, and measured the percentage of PD1-expressing BM cells. The frequency of PD1+ BM cells increased significantly from 6.9 to 12%, and the mean fluorescence intensity (MFI) of PD1 expression rose by approximately 43% after rmB7-H3 treatment (**Figure 6A**). Flow cytometry analysis further showed that PD1+ CD84+ GMPs in undifferentiated BM cultures were increased by nearly 3-fold in response to B7-H3, whereas this increase was abolished after GM-CSF-induced differentiation (**Figure 6B**). Consistently, rmB7-H3 induced more than a 2-fold increase in PD1 mRNA expression in sorted BM MDSCs (CD11b+ Gr1+) (**Figure 6C**), indicating that B7-H3 may play a regulatory role in the differentiation of PD1-expressing MDSCs at the progenitor level. To assess the role of PD-1 expression in myeloid cells in B7-H3-mediated myeloid cell differentiation, we next examined the impact of B7-H3 on myeloid cell-specific PD1-deficient cells from LysMCre-PD1 knockout (KO) mice. Ablation of PD1 in myeloid cells eliminated the rmB7-H3-induced increase in M-MDSCs compared with WT BM cells (0.98 in KO vs 2.1-fold in WT) (**Figure 6D**), and this was associated with significant reduction in the M-MDSC marker Nos2 mRNA (**Figure 6E**). In contrast, when PD1 was absent in myeloid cells, rmB7-H3 treatment increased MHCII+F4/80+ macrophage-like cells by approximately 1.8-fold (**Figure 6F**). MDSC progenitor GMPs have been identified as a key target of PD1 during myelopoiesis, and GMP accumulation is significantly reduced by PD1 ablation in the murine B16-F10 melanoma model (51). Together, these findings suggest that PD1 expression potentially in GMPs may be involved in B7-H3-skewing towards immunosuppressive M-MDSCs.

### 3.7 B7-H3 blockade inhibits MDSC expansion and BLM-induced lung fibrosis

**Figure 7:**
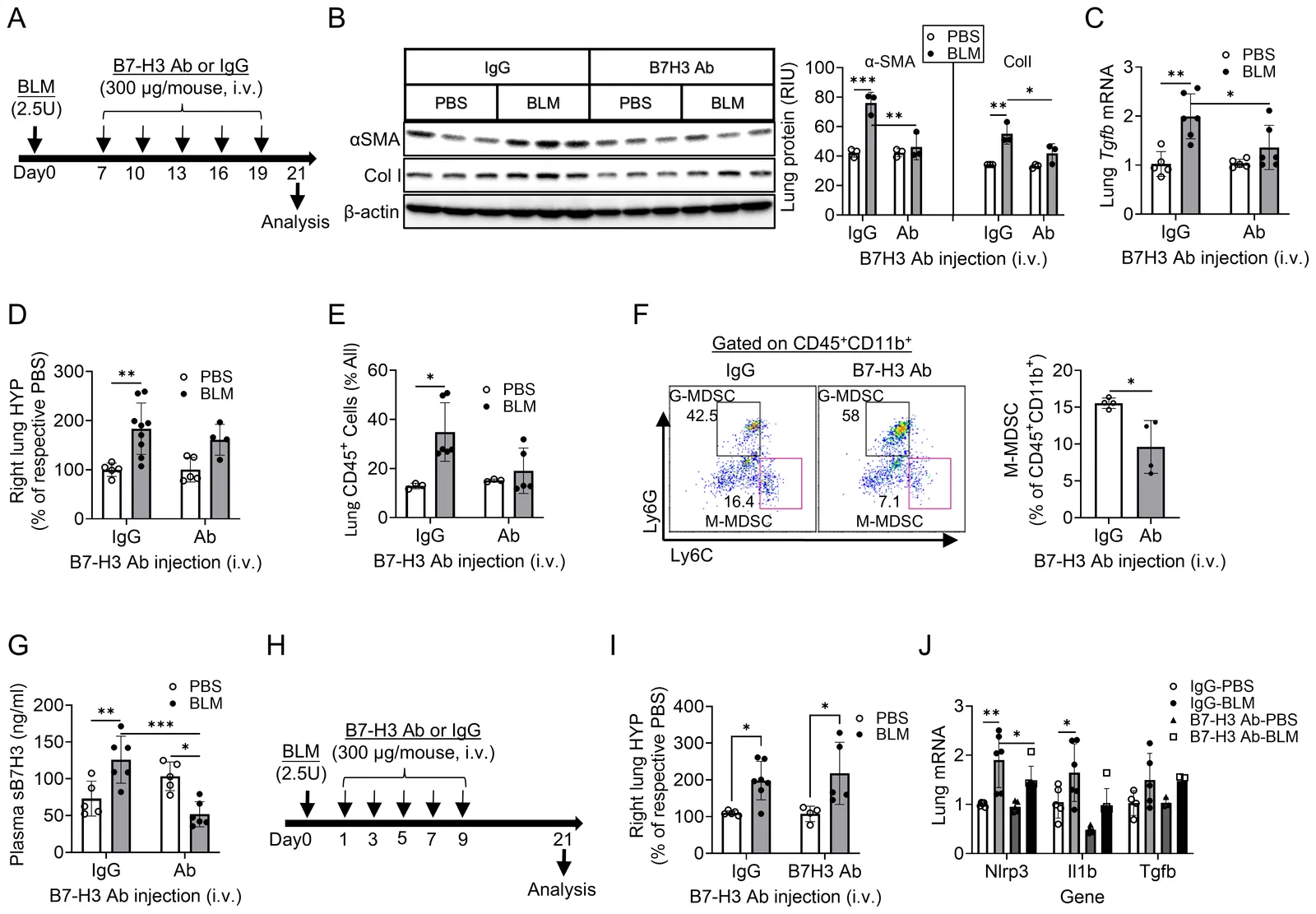
B7-H3 delayed antibody treatment reduces BLM-induced lung fibrosis and BM-derived M-MDSC accumulation. **(A)** BLM was administrated into 6–8 weeks old C57BL/6J WT female mice on day 0. Anti-B7-H3 blocking antibody **(Ab)** or IgG was injected intravenously starting on day 7 and given in five doses as indicated. Mice were euthanized on day 21 after BLM injection. **(B)** Lung tissue lysates were analyzed for α-SMA and collagen I protein levels by Western blotting with β-actin as a loading control. Representative western blots (left) and quantitative analysis relative unit to β-actin intensity (RIU, right) are shown. **(C)** Lung RNA was analyzed for TGFβ mRNA (Tgfb) by qPCR and is presented as fold change relative to WT controls. **(D)** Right lung homogenates were analyzed for hydroxyproline content. Lung single-cell suspensions were analyzed by flow cytometry for CD45^+^ cells **(E)** and for M-MDSC (CD45^+^ CD11b^+^ Ly6C^hi^ Ly6G^-^) in BLM groups **(F)**; a representative flow cytometry plot is shown. The pre-gating strategy for CD45^+^CD11b^+^ parental population is described in **Figure 1F**. **(G)** Plasma samples were analyzed for sB7-H3 by ELISA. **(H)** BLM was administrated on day 0. Anti-B7-H3 blocking Ab was injected intravenously between days 1 and 9 and given in 5 doses as indicated. **(I)** Right lung homogenates were analyzed for hydroxyproline content. N = 4–10 mice per group. At least two independent mice experiments were performed. **(J)** Lung RNA extracted on day 21 after BLM treatment was analyzed for Nlrp3, Il1b and Tgfβ mRNA by qPCR, and data are shown as fold change relative to respective WT control are shown. N = 4-6. Student’s test was performed in 7F; one-way Anova analysis in 7B-7E, 7G, 7I, and 7J. *p 0.05, **p 0.01, ***p 0.005.

Our previous study indicates that systemic B7-H3 blockade with a B7-H3 blocking antibody at the early stage of fibrosis reduces BM-derived MDSC influx and lung inflammation during BLM-induced lung fibrosis (20). To investigate whether B7-H3 blockade has a therapeutic effect on established fibrosis, B7-H3 blocking antibody was administered 7 days after BLM installation, as shown in **Figure 7A**. The survival rates for BLM-treated mice were 80% in both IgG and B7-H3 antibody groups (**Supplementary Figure 6A**). B7-H3 antibody treatment at this time point significantly reduced fibroblast activation and differentiation, as indicated by decreased lung α-SMA and collagen I levels after BLM treatment compared with IgG treated controls (**Figure 7B**). BLM-induced TGFβ was also eliminated by B7-H3 antibody treatment (**Figure 7C**). As expected, lung collagen deposition, assessed by hydroxyproline content, was increased approximately 2-fold by BLM treatment in the control mice; this increase was significantly attenuated in the B7-H3 antibody-treated group (**Figure 7D**). BLM-induced influx of BM-derived CD45+ cells into the lung was clearly reduced in mice treated with B7-H3 antibody (**Figure 7E**), as was the percentage of BM-derived M-MDSCs, but not G-MDSCs (**Figure 7F**). Consistent with previous findings in human IPF and in the BLM model of lung fibrosis (18, 20), we observed that sB7-H3 levels increased in mouse plasma following BLM treatment; this BLM-induced rise was markedly diminished by B7-H3 antibody blockade, falling to levels even lower than baseline in the B7-H3 antibody group (**Figure 7G**). Five doses of B7-H3 antibody administered between days 1–9 after BLM treatment (**Figure 7H**) failed to reduce lung fibrosis, shown by the comparable BLM-induced hydroxyproline content between the IgG and B7-H3 antibody groups (**Figure 7I**), despite a decrease in lung inflammation as shown in **Figure 7J**. In addition, early-stage B7-H3 blockade failed to alter BLM-induced plasma sB7-H3 levels in the B7-H3 antibody-treated group compared to the IgG-treated group (**sFigure 6B**). These findings indicate that therapeutically timed B7-H3 blockade reduces established lung fibrosis and is associated with decreased plasma sB7-H3 levels.

### 3.8 Increased B7-H3^+^ cells in patients with IPF are positively correlated with peripheral circulating CD4^+^CD25^+^ T cells

**Figure 8:**
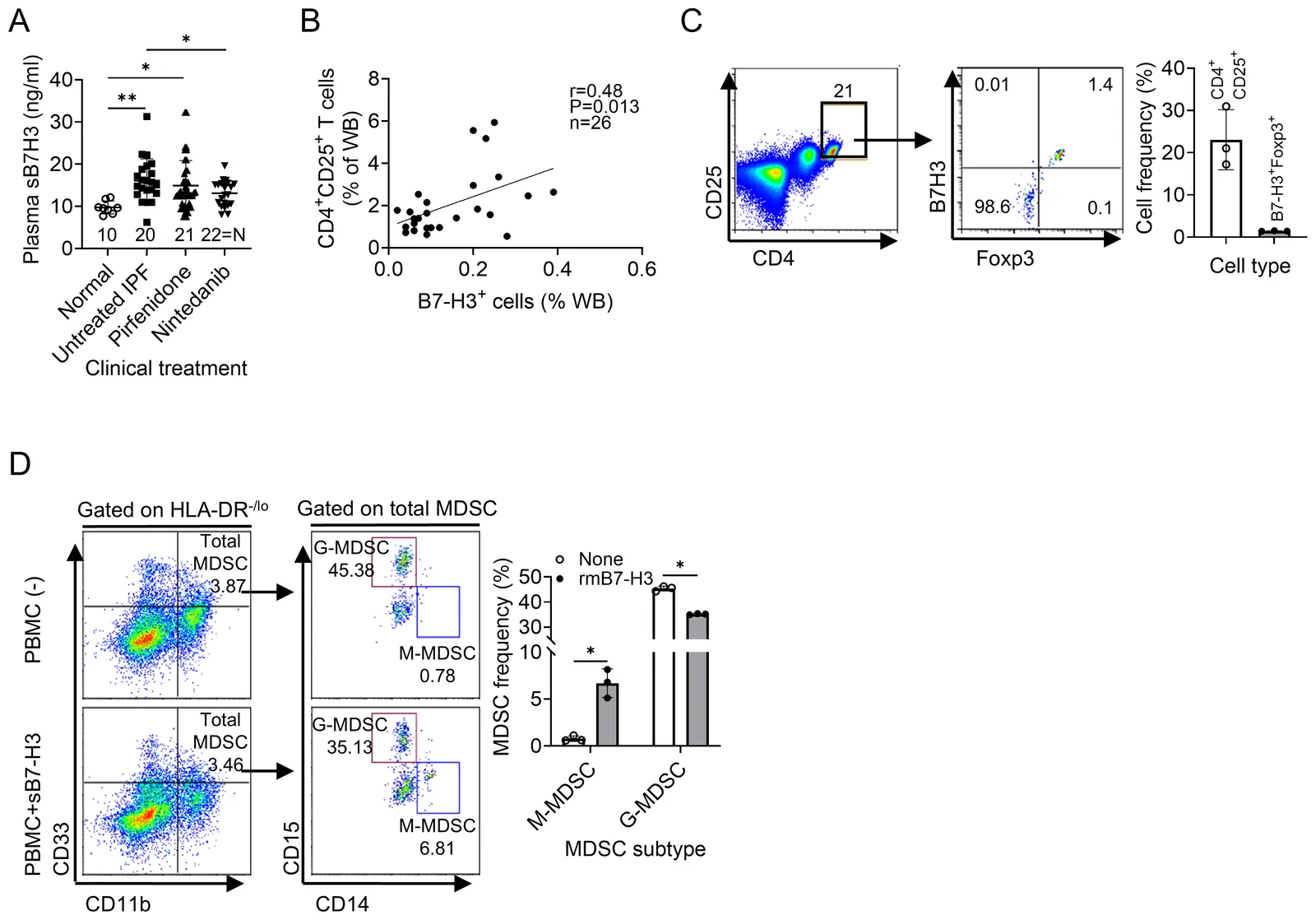
B7-H3 is increased in patients with IPF. **(A)** Plasma sB7-H3 levels in IPF patients and healthy subjects were measured by ELISA. Numbers below the data points indicate the number of subjects in each group. **(B)** White blood (WB) cells from peripheral blood of IPF patients were analyzed by flow cytometry for B7-H3^+^ and CD4^+^CD25^+^ T cells. The linear regression & Pearson’s correlation analyses were performed using GraphPad Prism 9.5.1. **(C)** Three PBMC samples from the same subjects as in **(B)** were analyzed for CD4^+^CD25^+^ T cells, and their Foxp3 and B7-H3 expression by flow cytometry. **(D)** In a pilot study, human PBMCs isolated from peripheral blood samples of two IPF patients were pooled, plated in 6-well plates, and treated with 2μ g/ml rhB7-H3 for 72 hours. Human MDSCs were then analyzed by flow cytometry using the gating strategy shown in **Supplementary Figure 7** in the supplemental data; a representative flow cytometry plot set and quantitative data are shown. Two independent experiments were performed with N = 3 per group in each experiment. Student’s test was performed in 8C; linear regression and Pearson correlation in 8B; one-way Anova analysis in 8A and 8D. *p 0.05, **p 0.01, ***p <0.005.

Finally, we examined plasma sB7-H3 levels and the peripheral blood frequency of B7-H3+ cells and CD4+ CD25+ T cells in patients with IPF to assess clinical relevance. Patients with IPF were stratified according to their clinical treatments. As expected, plasma sB7-H3 levels were elevated in all enrolled patients with IPF, but were lower in patients treated with Nintedanib, and not Pirfenidone, compared with untreated patients (**Figure 8A**). The frequency of B7-H3+ cells was positively correlated with CD4+ CD25+ T cells in peripheral blood sample from patients with IPF (**Figure 8B**). Interestingly, a subset of Foxp3-expressing CD4+ CD25+ T cells also express B7-H3, although this represented only a small fraction (approximately 1.4%) of the CD4+CD25+ Tregs population (**Figure 8C**). Our previous study showed that the frequency of M-MDSC, but not G-MDSC, is significantly lower in Nintedanib- or Pirfenidone-treated patients with IPF than in untreated patients (20). To determine whether this reduction M-MDSC is associated with decreased sB7-H3 after Nintedanib treatment, we further assessed the effect of B7-H3 on MDSC induction *in vitro* using PBMCs from two patients with IPF in a pilot study. Recombinant human B7-H3 (rhB7-H3) treatment significantly increased M-MDSC frequency, from 0.78% in untreated samples to 6.8% in treated samples, without a corresponding increase in G-MDSC (**Figure 8D**) and gating strategy is shown in **Supplementary Figure 7**. Given the limited number of human blood samples included, future studies including PBMC samples from a larger cohort will be warranted to strengthen and validate these preliminary findings and provide more clinically relevant evidence. These findings suggest that B7-H3 contributes to an enhanced immunosuppressive environment in IPF, including increase in CD4+CD25+ T cells. Given the positive correlation between circulating B7-H3+ cells and plasma sB7-H3 (18), sB7-H3 levels in patients with IPF may serve as a biomarker reflecting disease stage and immunosuppressive status in pulmonary fibrosis.

## 4 Discussion

BM-derived MDSCs are recruited and expanded in cancer, infection, inflammation, and fibrosis such as IPF, and their accumulation correlates with disease severity (1, 3, 12, 18–20). Previous reports demonstrate that B7-H3 is significantly elevated during pulmonary fibrosis: patients with IPF have increased plasma sB7-H3 and B7-H3+ circulating cells that negatively correlate with lung function, and MDSC expansion is associated with B7-H3+ cell numbers and disease severity. Functionally, sB7-H3 can recruit/activate BM M-MDSCs and enhance MDSC immunosuppression of T-cell proliferation. *In vivo*, B7-H3 blockade reduced BM MDSC recruitment to the fibrotic lung (18, 20, 36, 37). Despite these important findings, the underlying mechanisms remained unexplored, including: how B7-H3 drives expansion and recruitment of BM-derived M-MDSCs; specific role of sB7-H3 in the development of pulmonary fibrosis; the mechanism by which B7-H3 enhancement of MDSC suppression of T cell proliferation is mediated; and whether B7-H3 plays cell-type specific functions.

To begin addressing these gaps, we analyzed single-cell RNA-sequencing data from the IPF Cell Atlas and found that lung fibroblasts and myofibroblasts are major sources of B7-H3 in IPF lung (20). In addition, our prior work shows that lung fibrotic fibroblasts have higher B7-H3 mRNA expression than controls (18). These findings prompted us to investigate the role of fibroblast-derived B7-H3 in the context of BLM-induced pulmonary fibrosis. Our data show that fibroblast-specific B7-H3 deficiency reduced lung fibrosis and was accompanied by decreased BM-derived MDSC influx, and increased MHCII+ F4/80+ cells *in vivo*. Additionally, B7-H3 deficiency in myeloid cells altered M-MDSC and myeloid differentiation but did not ameliorate fibrosis, suggesting diverse cell-type specific roles of B7-H3 during disease progression. In a BLM model of lung fibrosis, the role of fibroblast-specific B7-H3 deficiency in reducing fibrosis was consistent with the effects of B7-H3 blockade after fibrosis onset, corresponding to the abundance of fibroblasts and myofibroblasts at later stages of the fibrotic response. Future work will determine whether the different outcomes reflect timing-dependent biology. Moreover, our data indicate that sB7-H3 cleaved from activated lung fibroblasts, perhaps by MMPs, can promote BM MDSC chemotaxis through Tlt2 expressed on the MDSCs *in vitro*. Due to the use of the broad-spectrum MMPI, GM6001, future studies will be needed to determine whether fibroblast-derived sB7-H3 directly mediates these effects.

In support of our findings, a related study in a human breast cancer model identifies a carcinoma-associated fibroblast subset (CAF-S1) that recruits CD25+ Foxp3+ Tregs via B7-H3 expression and fosters an immunosuppressive tumor microenvironment. Notably B7-H3-expressing CAF-S1 fibroblasts express fibroblast activation protein α1 (FAP) and α-SMA, suggesting that they represent a myofibroblast population. Silencing of B7-H3 in CAF-S1 fibroblasts significantly decreases their ability to expand CD25+ Foxp3+ Tregs (52). This study suggests that anti-B7-H3 therapy might also target CAF-S1 cells, thereby enhancing antitumor immunity by reducing CAF-S1-mediated immunosuppression. Together, these findings support a model in which fibroblast-derived sB7-H3 acts as a signaling molecule from injured or fibrotic lungs to the BM, promoting early-stage MDSC development and differentiation from progenitor cells. Fibroblast-derived sB7-H3 may reinforce the immunosuppressive phenotype of differentiated myofibroblasts, amplifying immunosuppressive networks in fibrosis and cancer (53). The observation that fibrosis reduction was greater in the fibroblast specific B7-H3 cKO than in the myeloid specific B7-H3 KO mice provides further evidence that fibroblast-derived sB7-H3 plays a central role in MDSC generation during chronic lung remodeling.

Having established fibroblasts as one of the major sources of B7-H3, we investigated the regulatory role of B7-H3 in MDSC generation and activation with the goal to elucidate the mechanisms underlying B7-H3 modulation of MDSC differentiation from their progenitors, potentially PD1-expressing-GMPs. Our results show that specific PD1 ablation in LysM-expressing myeloid cells, including GMPs, eliminates B7-H3-induced M-MDSC differentiation and restores MHCII+ F4/80+ cell differentiation, indicative of myeloid cell maturation. B7-H3 deficiency reduces PD1+ GMPs expansion in the BM after BLM injury. Thus, impaired differentiation at the site of injury (54, 55) and BM recruitment can play a role in MDSC differentiation. There is evidence that MDSC generation occurs locally at target tissues, such as the lung, where immature or progenitor myeloid cells migrate in response to injury or inflammatory stimuli (20, 36, 56, 57). Additionally, our findings are consistent with a recent study showing that MDSC generation from GMPs can result from blockade of GMP differentiation into mature myeloid cells in a murine model of mammary cancer. In that study, treatment with dihydroorotate dehydrogenase inhibitor, BRQ, diminishes MDSC production from early-stage myeloid progenitors GMPs, while enhancing myeloid maturation. In addition, BRQ modulates MDSC suppressive capacity and CD84 expression when combined with PD1-based immunotherapy (39). Furthermore, dual blockade of PD1 and B7-H3 in mice further augments anti-tumor immunity, underscoring the therapeutic potential of B7-H3-PD1 combination strategies in reducing MDSC numbers in the tumor microenvironment. In the context of fibrosis, we show that in patients with IPF, nintedanib, but not pirfenidone, reduces plasma sB7-H3 levels highlighting the role of B7-H3 as a potential biomarker and therapeutic target for fibrosis. Clinically, B7-H3 expression in patient-derived NSCLC samples correlates strongly with non-responsiveness to anti-PD1 therapy underscoring the role for MDSC in driving immunosuppression (58). Collectively, these data support a model in which B7-H3 drives MDSC differentiation from their progenitors at early stage under pathogenic conditions both locally and in BM.

Having explored how MDSCs arise, we next considered how MDSC function. MDSCs are a highly heterogeneous population with M-MDSC and G-MDSC subpopulations exhibiting distinct immunosuppressive capacities (6, 26, 27, 50). Furthermore, because of their heterogenicity, cell surface markers are not sufficient to identify the respective populations. In order to validate MDSCs and establish their functionality we assessed their capacity to suppress T cell proliferation. Relative to G-MDSCs, M-MDSCs have more potent immunosuppressive activity, which correlates with CD84 expression; thus CD84hi-MDSCs exhibit a greater effect on T cell suppression compared with CD84lo MDSCs (50). Our group and other studies have shown that B7-H3 appears to exert a predominant effect on M-MDSCs, potentially due to its induction of CD84 expression (20, 39, 50). Indeed, we found that CD84 blockade markedly reduces B7-H3-induced M-MDSC generation and functionally reverses their ability to inhibit T cell proliferation. Given that CD84 is expressed on nearly all M-MDSCs, but only ~8% G-MDSCs (20), CD84+ M-MDSCs likely represent the immunosuppressive subpopulation of MDSCs. Furthermore, our findings suggest that CD84 might functionally mediate B7-H3-enhanced MDSC immunosuppression of T cell proliferation, in addition to serving as a suppressive marker on M-MDSCs, although further investigation is needed to fully elucidate B7-H3-CD84 signaling.

**Figure 9:**
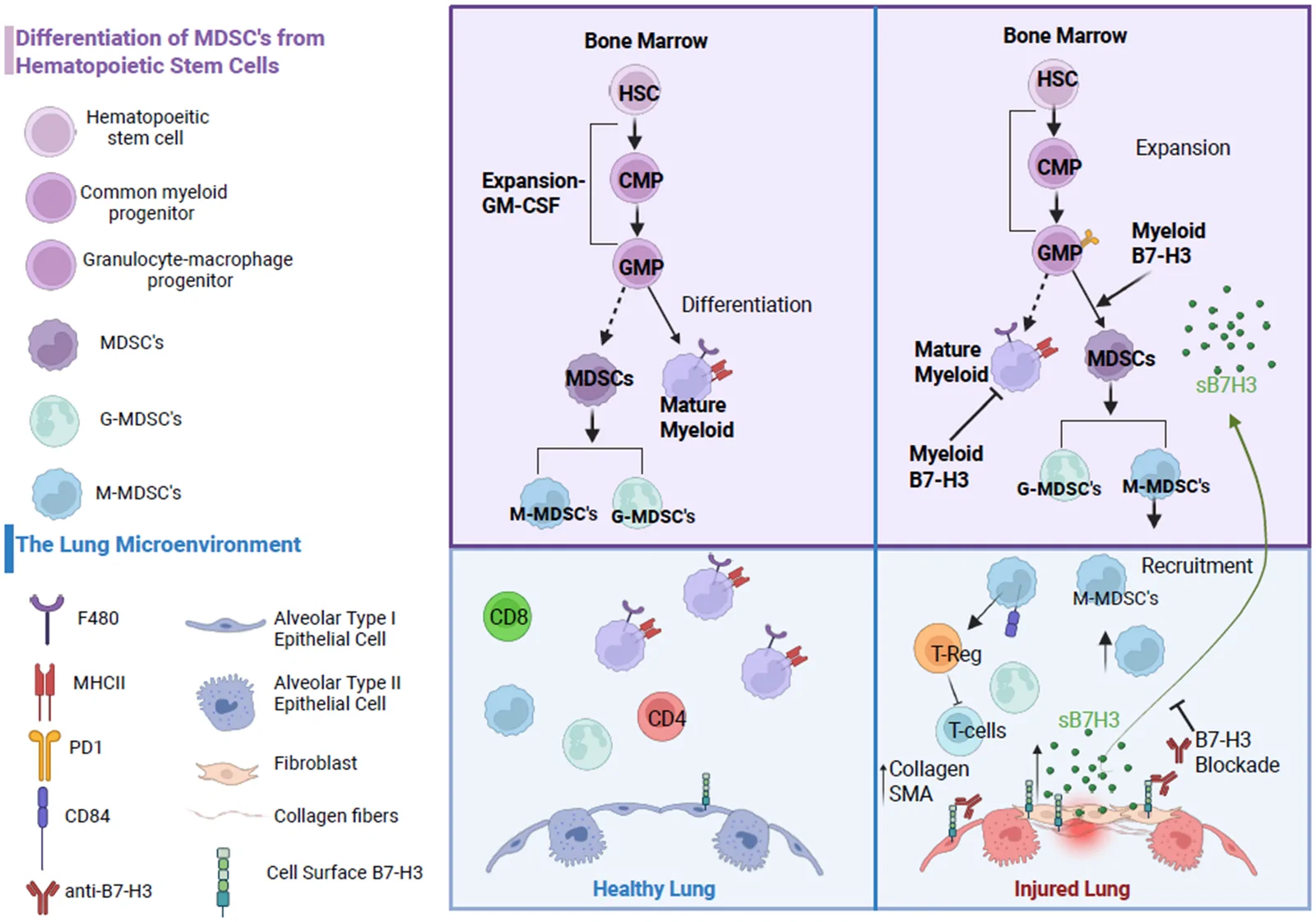
Schematic diagram illustrating the role of fibroblast and myeloid-derived B7-H3 in MDSC differentiation. Hematopoietic progenitor cells (HPCs) proliferate and differentiate to common myeloid progenitors (CMPs) and subsequently into granulocyte/monocyte progenitors (GMPs) under GM-CSF stimulation. During homeostatic conditions, GMPs differentiate into mature myeloid cells such as F4/80^+^MHCII^+^ macrophage-like cells, with relative limited generation of immature MDSCs. During fibrosis, lung fibroblasts secrete sB7-H3, which signals to BM progenitors GMP to upregulate PD1 expression and acquire an immunosuppressive phenotype, thereby skewing differentiation toward MDSCs rather than mature myeloid cells. Additionally, B7-H3 promotes expansion and recruitment of M-MDSCs to the fibrotic lung and enhances their immunosuppressive effects on T cell activation and proliferation through CD84 signaling. This model proposes that B7-H3 orchestrate an immunosuppressive cellular network that sustains pulmonary fibrosis. Created in BioRender. Gonzalez De Los Santos, F (2026). https://BioRender.com/n2l5vfh.

Taken together, our findings provide new insights into the role of B7-H3 in regulating MDSC differentiation during lung fibrosis. B7-H3-induced expansion of MDSCs during fibrosis may reflect a shift in myeloid differentiation toward MDSCs at the progenitor level, rather than only accumulation. Soluble B7-H3 released from fibrotic lung fibroblasts may contribute to driving MDSC differentiation from PD1-expressing progenitors, while inhibiting differentiation toward macrophage-like cells; moreover, sB7-H3 exhibits chemotactic activity toward BM-MDSCs, which is potentially mediated by its putative receptor, Tlt2. While previous studies have identified CD84 as an immunosuppressive marker of M-MDSCs, we now reveal its functional role in mediating B7-H3-enhanced MDSC immunosuppression of T cell proliferation. Lastly, although myeloid B7-H3 promotes MDSC differentiation and blocks macrophage-like cell differentiation, potentially at the early progenitor stage, we find that fibroblast-derived sB7-H3 is more critical in regulating fibrosis. This is consistent with our results, which show that, compared with early blockade of B7-H3, delayed blockade is more effective, corresponding to a stage at which activated fibroblasts are abundant, suggesting that targeting B7-H3 may represent a novel therapeutic strategy to eliminate MDSCs in pulmonary fibrosis.We summarize these findings in a schematic illustration of the proposed actions of B7-H3 in lung fibrosis (**Figure 9**).

In summary, our studies reveal novel mechanisms by which B7-H3 regulates the differentiation and activation of MDSCs while inhibiting normal myeloid cell differentiation. Because of the phenotypic overlap between MDSCs and mature myeloid cells, the selective elimination of MDSCs remains challenging. However, our findings identify B7-H3 as a potential therapeutic target whose inhibition ameliorates pro-fibrotic myeloid polarization, resulting in the reduction of established fibrotic lesions.

## Data Availability

The original contributions presented in the study are included in the article/**Supplementary Material**. Further inquiries can be directed to the corresponding authors.
